# Presence of fetal microchimerisms in the heart and effect on cardiac repair

**DOI:** 10.3389/fcell.2024.1390533

**Published:** 2024-08-05

**Authors:** Vicente Llorente, Marina López-Olañeta, Elena Blázquez-López, Elena Vázquez-Ogando, Magdalena Martínez-García, Javier Vaquero, Susana Carmona, Manuel Desco, Enrique Lara-Pezzi, María Victoria Gómez-Gaviro

**Affiliations:** ^1^ Instituto de Investigación Sanitaria Gregorio Marañón (IiSGM), Madrid, Spain; ^2^ Centro Nacional de Investigaciones Cardiovasculares (CNIC), Madrid, Spain; ^3^ Servicio de Ap. Digestivo del HGU Gregorio Marañón, Instituto de Investigación Sanitaria Gregorio Marañón (IiSGM), Madrid, Spain; ^4^ Centro de Investigación Biomédica en Red de Salud Mental (CIBERSAM), Madrid, Spain; ^5^ Centro de Investigación Biomédica en Red de Enfermedades Hepáticas y Digestivas (CIBEREHD), Madrid, Spain; ^6^ Departamento de Bioingeniería, Universidad Carlos III de Madrid, Leganés, Spain

**Keywords:** chimerism, feto-maternal microchimerism, myocardial infarction, echocardiography, animal models

## Abstract

Multiple complex biological processes take place during pregnancy, including the migration of fetal cells to maternal circulation and their subsequent engraftment in maternal tissues, where they form microchimerisms. Fetal microchimerisms have been identified in several tissues; nevertheless, their functional role remains largely unknown. Different reports suggest these cells contribute to tissue repair and modulate the immune response, but they have also been associated with pre-eclampsia and tumor formation. In the maternal heart, cells of fetal origin can contribute to different cell lineages after myocardial infarction. However, the functional role of these cells and their effect on cardiac function and repair are unknown. In this work, we found that microchimerisms of fetal origin are present in the maternal circulation and graft in the heart. To determine their functional role, WT female mice were crossed with male mice expressing the diphtheria toxin (DT) receptor. Mothers were treated with DT to eliminate microchimerisms and the response to myocardial infarction was investigated. We found that removal of microchimerisms improved cardiac contraction in postpartum and post-infarction model females compared to untreated mice, where DT administration had no significant effects. These results suggest that microchimerisms play a detrimental role in the mother following myocardial infarction.

## 1 Introduction

Pregnancy carries a series of biological adaptations aimed at ensuring a healthy gestation, birth and postpartum. These include a process where cells are exchanged between fetus and mother; thus, the fetus contributes cells to several organs of its mother, including liver, kidneys, and brain, forming microchimerisms (Boddy et al., 2015; Cómitre-Mariano et al., 2022). The same applies to the mother’s cells, which migrate into the fetus’ own developing organs and form maternal microchimerisms in turn, although seemingly at lower rates (Loubière et al., 2006; Fujimoto et al., 2021). This bidirectional flow of cells increases as gestation progresses and is more pronounced in the latter half of the pregnancy (Adams Waldorf et al., 2010), and the method of delivery can affect the rate of occurrence, with Cesarean delivery increasing its probabilities compared to uncomplicated vaginal deliveries (Shree et al., 2019). Placental disfunction (Fjeldstad et al., 2023b), and mother’s age (Roh et al., 2017) positively correlate with the occurrence of microchimerisms as well.

While some of these cells are cleared by the mother’s immune system after birth, many persist for decades (Bianchi et al., 1996; Evans et al., 1999; Maloney et al., 1999; Bayes-Genis et al., 2005). Transference of fetal cells to the mother is conserved from the evolutionary point of view in placental mammals (Gammill and Nelson, 2010), including rodents, cattle (Turin et al., 2007), goats (Gash et al., 2019), and pigs (Wang et al., 2022). These chimeric cells have been reported in the liver (Johnson et al., 2002; Guettier et al., 2005), heart (Kara et al., 2012; Kara et al., 2012; Lintao et al., 2023), brain (Tan et al., 2005; Chan et al., 2012), bone marrow (O’Donoghue et al., 2004), kidney (Florim et al., 2015), lung, and spleen (Rijnink et al., 2015).

Despite the consensus on the existence of these fetal microchimerisms, their functional effect is far from being understood; especially in the long term after pregnancy (Boddy et al., 2015). It remains unclear whether the effect of these microchimerisms is beneficial or detrimental. It has been proposed that cells coming from the fetus have an active role in the mother’s immunomodulation, increasing the immunological tolerance to paternal antigens with the aim of increasing the tolerance to the fetus itself (Kinder et al., 2017). The presence of fetal cells in maternal organs is associated with an increase in CD4^+^ regulatory lymphocytes (Treg) with immunosuppressive action (Aluvihare, Kallikourdis, and Betz, 2004), and has been tentatively linked to reduced risk of brain cancer (Kamper-Jørgensen et al., 2022) and better clinical outcomes on COVID-19 (Cirello et al., 2023). In the heart, fetal microchimerisms have been linked to the mitigation of the inflammatory response in experimental autoimmune myocarditis (Ribeiro et al., 2022). Fetal cells may also participate in tissue repair, contributing to the formation of fibrotic scar and angiogenesis (Nassar et al., 2012; Alkobtawi et al., 2022; Sbeih et al., 2022), and could also play a role in psychology and mother-child bonding (Álvarez et al., 2023); this last role may be included in the structural and functional changes experienced by maternal brains during pregnancy (Hoekzema et al., 2017; Barba-Müller et al., 2019).

On the other hand, fetal microchimerisms could increase the mother’s susceptibility to auto-immune diseases (Nelson, 2012; McCartney et al., 2023) as well as endocrine pathologies (Fugazzola, Cirello, and Beck-Peccoz, 2012). They have also been associated with preeclampsia, miscarriages, premature births, and fetal growth restrictions (Leung et al., 1998; Al-Mufti et al., 2000; Gammill et al., 2013; Peterson et al., 2013). Poor glucose control and placental dysfunction in diabetic pregnancies correlate with an increase in the rate of fetal microchimerisms (Fjeldstad et al., 2023a). The presence of microchimerisms has been reported in the adult heart in both human and mice and has been mainly associated with changes in immune cell populations, in line with their proposed immunomodulatory role. Furthermore, transdifferentiation into different maternal cardiac cell populations has been proposed and their number increases following myocardial infarction (Kara et al., 2012). However, the functional role of these cells has not been specifically explored.

Myocardial infarction due to blockage of a coronary artery causes cardiomyocytic death through the lack of oxygen and nutrient supply, resulting in a reduction of the contractile capacity of the heart. The extremely limited capacity for regeneration possessed by the heart prevents the proper replacement of dead cardiomyocytes. Myocardial infarction (MI) has a prevalence of 3% on adults over 20 years old in the United States, where it causes one out of every 7 deaths (Tsao et al., 2022). Estimates point to 580.000 new cases and 210.000 recurring cases every year in the US, with around 165.000 “silent” infarctions as well. Prevalence is slightly lower in women, although the value itself remains high (2.3%). The probability of death because of MI is higher in women than in men, partly because they tend to suffer these cases at an older age. While recent advances in treatment and diagnosis have brought down the mortality rates of MI, there is still no cure, and all available treatments are palliative only.

Myocardial infarctions taking place in late pregnancy and postpartum display a much higher recovery rate compared to other pathologies (Felker et al., 2000), which lead to the hypothesis that fetal cells somehow contribute to cardiac repair. Placenta-derived stem cells have shown the ability to develop cardiac and vascular lineages *in vitro*, and their administration in a myocardial infarction model resulted in reduced adverse remodeling and improved function (Vadakke-Madathil et al., 2019). Microchimerisms in the form of multipotent fetal cells have been shown to home in on injured areas of maternal hearts *in vivo*, and are capable of differentiating into endothelial cells, smooth muscle cells, and spontaneously beating cardiomyocytes *in vitro* (Kara et al., 2012; Kara et al., 2012). Although no organized sarcomeres were detected *in vivo*, an immature cardiac phenotype was on display instead. However, the actual functional role of these cells and whether they help or hinder the mother’s own response to injury have yet to be studied.

In this work, we investigated the functional contribution of fetal cells to the maternal heart in rodents, by evaluating the effects of fetal microchimerism reduction in an experimental model of myocardial infarction.

## 2 Materials and methods

### 2.1 Mice

All experimental procedures included in this project were carried out in conformity with European Union Directive 2010/63/EU and were approved by Hospital Gregorio Marañón’s Research Committee and Ethical Committee for Animal Experimentation (ES280790000087).

C57BL6/J mice were obtained from Jackson Laboratory for this experiment. Transgenic mice with an EGFP gene in their ROSA26 locus blocked by LoxP-flanked STOP fragment (B6; 129-Gt(ROSA)26Sortm2Sho/J, #004077) were crossed with transgenic Sox2-Cre mice (B6.Cg-Edil3Tg(Sox2-cre)1Amc/J, #008454). This resulted in offspring with EGFP expression in all cells derived from the epiblast during embryogenesis, as well as any tissue with constitutive Sox2 expression (ROSA26-EGFP-Sox2).

**FIGURE 1:**
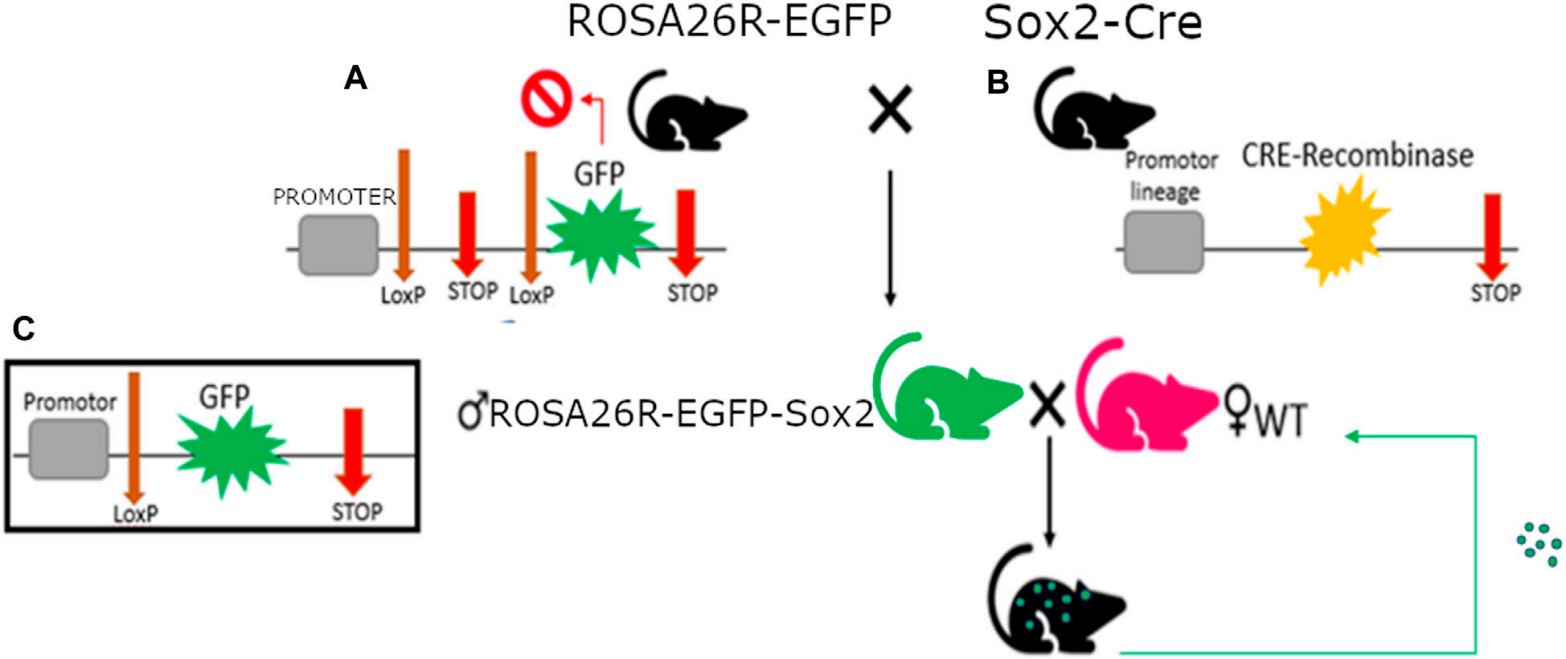
*Mus musculus* genotypes with single fluorescent protein expression used in this study. **(A)** RO-SA26R-EGFP genotype with construct detail, composed of a housekeeping promoter next a STOP codon flanked by LoxP sequences and followed by the GFP gene; the first STOP codon will be removed by the Cre-recombinase wherever it’s expressed, triggering GFP expression. **(B)** Sox2-Cre genotype, with Cre-recombinase expression in Sox2+ cells. **(C)** Rosa26R-EGFP-Sox2 male genotype, with constitutive expression of GFP in all Sox2+ cells.

Male ROSA26-EGFP-Sox2 mice were then crossed with WT mice to produce heterozygous offspring with similar Sox2-triggered EGFP expression in their organism (Figure 1).

### 2.2 Diphtheria toxin receptor (DTR) mice

For the functional study, male C57BL6/J mice were modified to express DTR in all their cells.

While a cross between DTR/DTR males and WT/WT females was attempted to guarantee WT/DTR genotype offspring (and thus full microchimerism clearance upon DT inoculation), breeding was unsuccessful, and heterozygotic males were used instead. Male heterozygotic WT/DTR mice were crossbred with female WT/WT mice, resulting in offspring with WT/WT and WT/DTR genotypes, which would likewise produce microchimerisms displaying both genotypes. Cells expressing DTR die after exposure to Diphtheria Toxin (DT), allowing WT/DTR genotype cells to be selectively eliminated through DT inoculation (200 ng, intraperitoneal injection).

This injection was carried out on female breeders 3 days after giving birth, in order to clear any WT/DTR microchimerisms present in their organism. Further administrations were performed at post-partum day 6 and day 9 in order to maximize FMc depletion, and the myocardial infarction model was carried out at post-partum day 10, 7 days after first DT injection, following previous reports of DTR + cell clearance on mouse heart (Cha et al., 2003; Akazawa et al., 2004).

### 2.3 Myocardial infarction pathological model

The myocardial infarction experimental model consisted on the ligation of the Left Anterior Descending coronary artery (LAD-ligation), as previously described (Villalba-Orero et al., 2021). Briefly, a week after DT injection, mice were anesthetized, intubated, and mechanically ventilated for the procedure. A left thoracotomy was performed between the third and fourth ribs, and the left descending artery was permanently ligated after pericardiectomy to induce a myocardial infarction.

### 2.4 Echocardiography

Transthoracic echocardiography was performed at three time points: before the LAD-ligation (baseline), and at days 3 and 28 after the LAD-ligation. This process was performed by an expert operator using a high-frequency ultrasound system (Vevo 2100, Visualsonics Inc.), in blinded conditions.

The parasternal long axis was analyzed at three levels (basal, mid, and apical) and all measurements were averaged over three consecutive cardiac cycles. LVEF and FS were calculated using the modified Quinone method, using the following formulas:

$$\text{LVEF}=\left({\text{LVIDed}}^{2}-{\text{LVIDes}}^{2}\right)/{\text{LVIDed}}^{2}$$

FS = (LVIDed−LVIDes)/LVIDes.

Where LVIDed is left ventricular internal diameter at end diastole and LVIDes is left ventricular internal diameter at end systole.

2D-guided M-mode of parasternal short-axis short (middle) was used to measure ventricle wall thickness.

### 2.5 Infarct size measurement

For the infarct size measurements, hearts were cut into 5 µm-thick slices along the transverse axis from apex to base with a microtome. Sections were stained using Masson’s trichrome (MTC) to study fibrosis, and midline infarct length was measured as previously described (Takagawa et al., 2007). Three measurements from three different slices of the same sample were averaged to obtain the % length value for each sample, and these values were averaged across all samples in the same group (N = 5 each).

### 2.6 Flow cytometry

For flow cytometry assays, blood samples (250 µL per animal) were collected under anesthesia on gestation day 20 and 30 days post-partum (N = 1) and stored in MiniCollect tubes with EDTA to prevent coagulation. An erythrocyte lysis protocol was carried out using Red Blood Cell Lysis solution (RBCL, cat#130-094-183, Miltenyi Biotec). Briefly, 100 µL of blood were mixed with 900 µL of RBCL and incubated for 10 min at room temperature (RT). Samples were centrifuged at 300 g for 5 min, and the supernatant was removed, obtaining an erythrocyte-free pellet.

Samples were analyzed using a MACSQuant Analyzer 16 flow cytometer (Miltenyi Biotec) and images were processed with the Kaluza software. Blood samples were analyzed without the use of antibodies, with direct detection of the intrinsic fluorescence showcased by the transgenic mice model used in this study.

For the myocardial sample assays, hearts were extracted from the animals (N = 2) after sacrifice through cervical dislocation and cut into three pieces each. No perfusion was included, and the samples were rinsed thoroughly with sterile phosphate-buffered saline (PBS 1x) afterwards to remove traces of blood and subjected to mechanical digestion with a syringe over a 40 μm cell strainer. FCM buffer (2% Fetal Bovine Serum and 0.1% sodium azide in PBS 1x) was added throughout the process. Samples were centrifuged for 5 min at 4°C at 1500RPM and resuspended, then stored at 4°C until they were analyzed with the flow cytometer. Myocardial cell samples were analyzed without the use of antibodies.

### 2.7 Statistical analysis

The statistical significance of the differences between group means was assessed through unpaired Student’s t-test or one-way ANOVA, as appropriate. In cases where ANOVA indicated inter-region variability, a multiple comparison was performed using the Tukey test. Data are presented as mean ± SD, and a *p* < 0.05 was considered statistically significant.

## 3 Results and discussion

### 3.1 Identification of fetal microchimerisms in circulating cells

**FIGURE 2:**
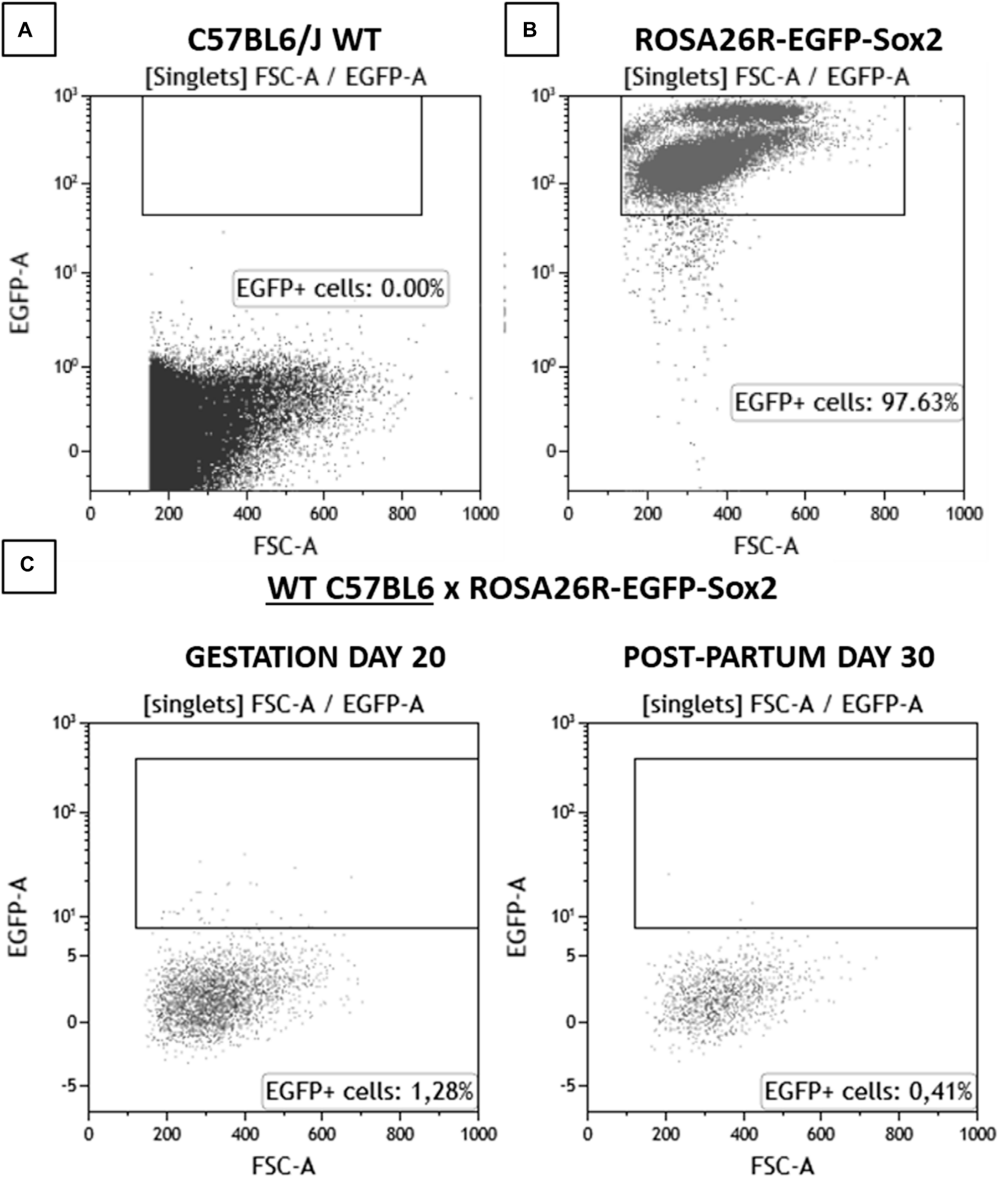
Flow cytometry analysis results for blood samples for **(A)** C57BL6/J WT, **(B)** ROSA26R-EGFP-Sox2, **(C)** C57BL6/J WT x ROSA26R-EGFP-Sox2 cross on gestation day 20 and 30 days post-partum. N = 1. Amount of detected EGFP + cells, corresponding to fetal-origin cells in systemic circulation.

To determine the presence of fetal microchimerisms, WT female mice were crossed with ROSA26-EGFP-Sox2 male mice, which express EGFP in all cells, including sperm. To verify the presence of fetal microchimerisms in maternal blood, blood samples were extracted from the WT female breeders at day 20 of pregnancy and 1 month post-partum, and the presence of EGFP + cells was analyzed using flow cytometry (Figure 2). Any fluorescence detected in blood would correspond to cells of fetal origin, as WT animals did not showcase any baseline fluorescence (Supplementary Figure S1). A slight increase in positive fluorescence cells (1.28%, compared to the baseline 0.08%) was detected at day 20, which would correspond to a moderate number of fetal cells having crossed into the maternal systemic circulation with the potential to form microchimerisms; this number was reduced at 30 days post-partum (0.41%), although the value still remained elevated compared to the baseline in control WT females. This observation would point to microchimerisms being present in the maternal circulation throughout pregnancy and remaining for at least 1 month after delivery, in accordance with previous studies which found even longer periods of permanence (Bianchi et al., 1996; Bayes-Genis et al., 2005; Kamper-Jørgensen et al., 2014).

**FIGURE 3:**
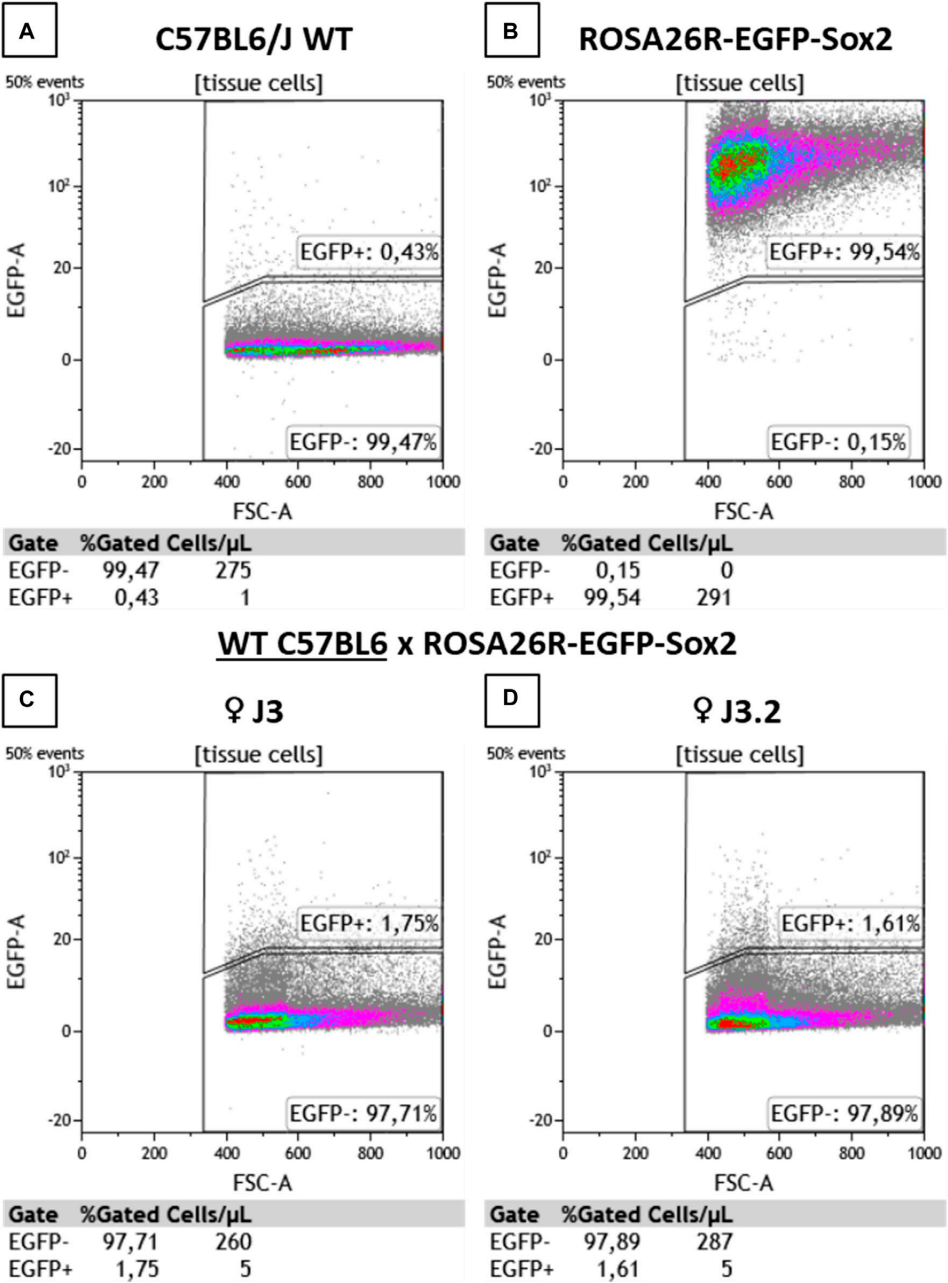
Flow cytometry analysis results for heart tissue samples. **(A)** C57BL6/J WT, **(B)** ROSA26R-EGFP-Sox2, **(C,D)** C57BL6/J WT x ROSA26R-EGFP-Sox2 cross maternal samples from **(C)** ♀ J3 and **(D)** ♀ J3.2. EGFP + cell detection results. J3 and J3.2 denote different animals from the same group. N = 2.

To determine the presence of microchimerisms in the heart, cells from myocardial samples were analyzed by flow cytometry (Figure 3). WT and ROSA26-EGFP-Sox2 mice were used as negative and positive controls, respectively. As expected, the WT mouse showed no EGFP + cells in the heart, while most cells in ROSA26-EGFP-Sox2 mice were EGFP+ (Figures 3A, B). Hearts from WT female mice crossed with ROSA26-EGFP-Sox2 male mice showed 1.6%–1.7% EGFP + cells (Figures 3C, D), indicating that fetal microchimerisms migrated and engrafted to the heart. EGFP + cells were also observed in maternal heart tissue samples through confocal microscopy, while there was no such fluorescence in the base WT animals (Supplementary Figures S2, S3).

### 3.2 Effect of microchimerism reduction on the myocardial infarction model

Since we had detected fetal microchimerisms in the heart, we next investigated whether these microchimerisms had any effect on cardiac function following myocardial infarction. For this purpose, WT female animals were crossed with male transgenic animals expressing diphtheria toxin receptor (DTR). Fetal microchimerisms present in maternal tissue were eliminated by treating the mothers with diphtheria toxin (DT), which induced the death of cells expressing the DTR (i.e., fetal microchimerisms).

DT was administered 3, 6, and 9 days post-partum to remove any cells expressing DTR. Myocardial infarction was induced through permanent ligation of the left descending coronary artery 10 days post-partum, and its presence was confirmed 3 days after surgery by echocardiography as a strong reduction in left ventricular ejection fraction (LVEF).

**FIGURE 4:**
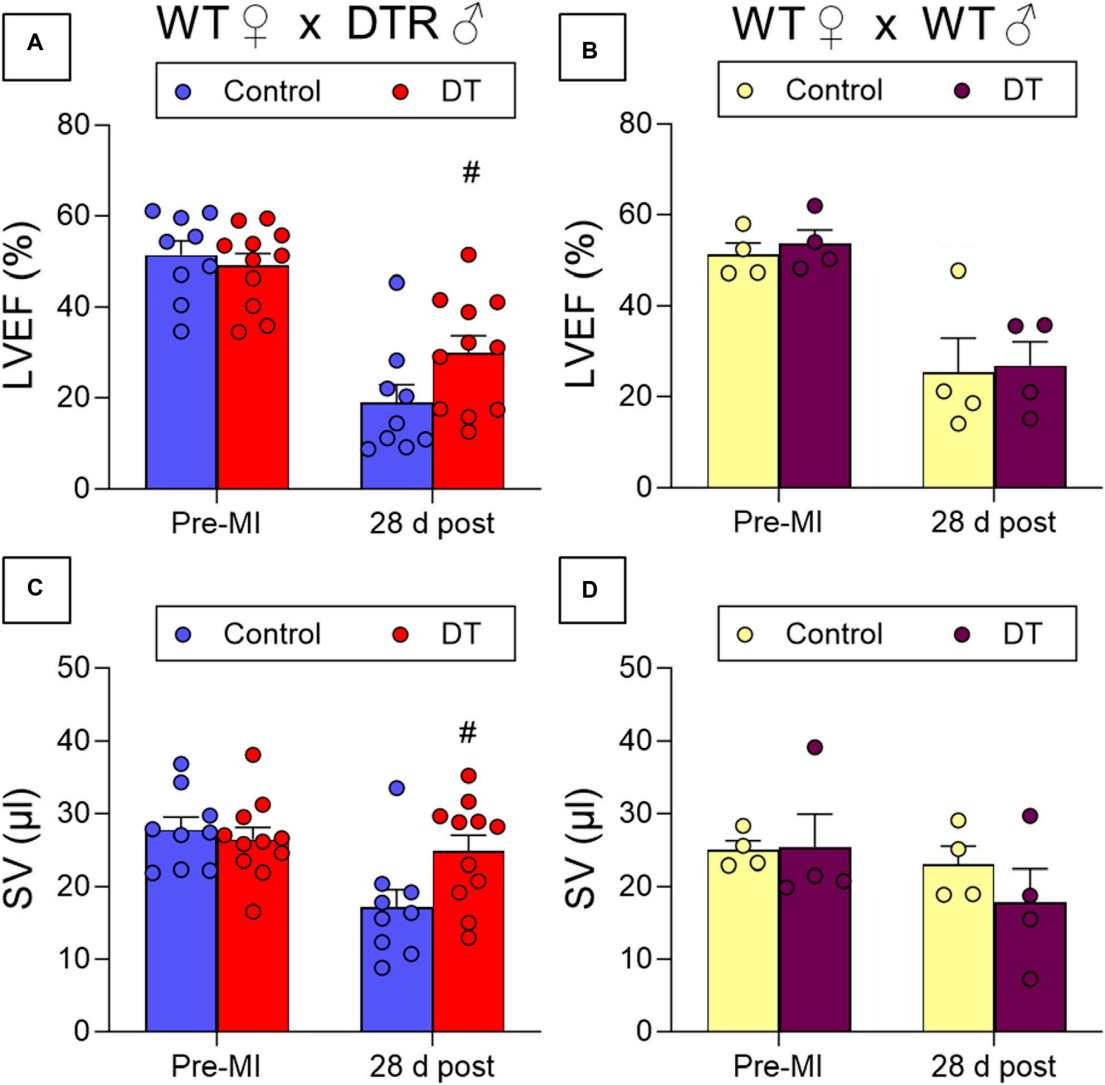
Effect of fetal microchimerisms reduction on cardiac function after myocardial infarction. WT female mice were crossed with DTR-expressing (N = 9/11) **(A,C)** or WT (N = 4) **(B,D)** male mice. Mice were treated (DT) or not (Control) with DT 3, 6, and 9 days post-partum and myocardial infarction was induced at 10 days by permanently ligating the left descending coronary artery. Functional parameters were measured using echocardiography before infarction and 28 days post-MI. Results show mean ± SEM. #*p* < 0.05 DT-treated vs. control mice, 2-way ANOVA followed by Šídák’s multiple comparisons test. LVEF: Left Ventricular Ejection Fraction, SV: Stroke Volume.

**FIGURE 5:**
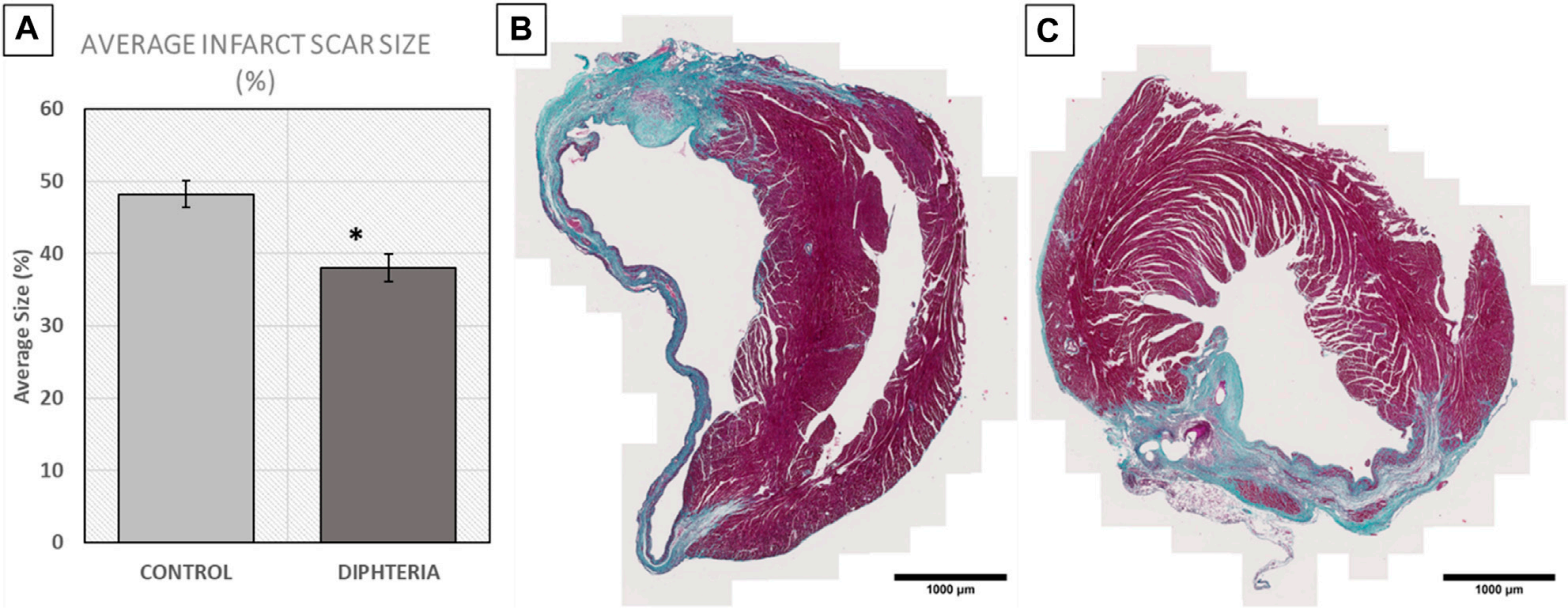
Myocardial infarct scar size comparison in control and diphtheria-treated (microQ-depleted) WTxDTR animals 28 days post-infarction. **(A)** Average infarct size % values. N = 5. *p < 0.05. **(B,C)** Representative images of Masson’s staining in **(B)** control and **(C)** diphteria-treated infarcted mouse heart samples. Scale bar = 1 mm.

DT treatment resulted in improved cardiac contraction, as evidenced by increased LVEF and stroke volume (SV) values 28 days post-MI in microchimerism-depleted mice compared to untreated mice. This effect following DT administration was not observed in WT mothers that had been crossed with WT males, suggesting that it was specific and caused by the depletion of DTR-expressing fetal microchimerisms in the maternal cardiac tissue (Figures 4A–D; Table 1). DT-treated animals also showcased a smaller average infarct length compared to its control group after histological study (Figure 5).

**TABLE 1:** Effect of fetal microchimerisms reduction on cardiac function after myocardial infarction. WT female mice were crossed with DTR or WT male mice. Mice were treated (DT) or not (Control) with DT 3, 6, and 9 days post-partum and myocardial infarction was induced at 10 days by permanently ligating the left descending coronary artery. Functional parameters were measured using echocardiography before infarction and 28 days post-MI. Results show mean values ± SE. *p < 0.05 infarcted vs. pre-MI, #*p* < 0.05 DT-treated vs. control, 2-way ANOVA plus Šídák’s multiple comparisons test.

Pre-MI	WT ♀ x DTR ♂		WT ♀ x WT ♂	
	Control	DT	Control	DT
EF (%)	51.36 ± 3.11	48.90 ± 2.64	52.80 ± 2.56	53.10 ± 3.05
SV (µL)	27.73 ± 1.77	26.04 ± 1.65	25.03 ± 1.25	25.22 ± 4.62
LVEDV (µL)	54.80 ± 3.24	53.48 ± 2.13	47.87 ± 3.71	46.83 ± 5.63
LVPWd (mm)	0.65 ± 0.02	0.71 ± 0.04	0.69 ± 0.09	0.65 ± 0.06
IVSd (mm)	0.65 ± 0.03	0.68 ± 0.07	0.59 ± 0.04	0.53 ± 0.05
n	9	11	4	4

28 days post-MI	Control	DT	Control	DT
EF (%)	18.89 ± 3.99*	29.86 ± 3.85*,^#^	25.34 ± 7.59 *	26.87 ± 5.23*
SV (µL)	17.20 ± 2.41*	24.85 ± 2.15^#^	23.02 ± 2.49	17.81 ± 4.64*
LVEDV (µL)	101.83 ± 9.00*	90.03 ± 6.54*	105.26 ± 18.23*	71.35 ± 20.52
LVPWd (mm)	0.75 ± 0.04	0.64 ± 0.04^#^	0.76 ± 0.16	0.72 ± 0.06
IVSd (mm)	0.77 ± 0.05*	0.65 ± 0.01^#^	0.53 ± 0.08	0.59 ± 0.05
n	9	11	4	4

EF, ejection fraction; LVEDV, left ventricular end diastolic volume; LVPWd, left ventricular posterior wall in diastole; IVSd, interventricular septum in diastole; n, number of mice analyzed.

While previous research from Kara et al. had pointed at fetal microchimerisms not only being present in maternal heart tissue but also specifically homing in on injured areas, there was no clear evidence of any effects on tissue repair, beneficial or detrimental. The capability of differentiation into cardiac cell lineages was demonstrated *in vitro* but not *in vivo*, where chimerisms just formed disorganized and undifferentiated structures and remained stuck. The result of this not only failed to support the hypothesis of fetal cells being able to aid in maternal injury repair and provide benefits, but it directly opposed such a notion. Indeed, the deletion of microchimerisms in female breeders undergoing myocardial infarction was associated with improved contractile capacity compared to mice in which microchimerisms were retained.

The reported lack of proper differentiation and structure *in vivo* may be the cause of this pernicious effect. Microchimerisms may be unable to mount a proper response to the damage and complete repairs to the tissue, while obstructing the mother’s own response. This situation would be more likely to occur in the natural environment, as opposed to previous reports where the specific positive selection for cells with the desired traits and the right environments may have ensured a higher efficiency and likelihood of an effective response. On the other hand, microchimerism-depleted females would rely exclusively on their own cells and regenerative processes, which would have a higher success rate and recover a higher degree of function after injury.

It should be noted that this depletion was not complete, as some fetal microchimerisms remained in the hearts because they did not express the DTR receptors; as such, one hypothesis would suggest that the process of cell death experienced by the DTR + microchimerism population could be triggering a proliferative and reparative response of the DTR-population in turn, which would prime them for a more efficient reparative action after MI, compared to the more extensive but inactive population of fetal microchimerisms present in non-depleted maternal hearts.

It is also worth pointing out that this study did not include any significant characterization of the cell populations involved in these dynamics; the overall size range of the cells detected through FC would potentially match a myocytic profile, as opposed to other cell types such as lymphocytes, but this doesn’t constitute definitive evidence of any specific cell type being favored by the fetal microchimerism population in the maternal heart. Likewise, the possibility of contamination of the heart samples from circulating cells present in traces of blood cannot be completely ruled out, with the potential skewing of the results regarding density and type of FMc present in cardiac tissue. Thus, it would be interesting to include that kind of effort in any future research on this topic, to better understand what is actually happening with the fetal microchimeric cells and the nature of whatever specific activity they are carrying out in a pathological context. For example, the immune system is involved in the response to MI, and as such it is possible that cells of fetal origin presenting an immune system phenotype could be taking part in that response and even resulting in such a negative effect as it may be inferred from the results of this study.

## 4 Conclusion

The presence of fetal-origin cells in the maternal organism in the shape of microchimerisms has been extensively reported, but the actual beneficial or detrimental effect they may have is still unclear. The present study confirmed the presence of cells of fetal origin in the mother’s heart and systemic circulation. Contrary to expectations, loss of microchimerisms resulted in improved cardiac contractility following myocardial infarction. While fetal cells are likely to hold the potential to differentiate into the required cell lineages and structures and aid in maternal wound repair, whether they are able to do so in normal circumstances and in all cases remains to be confirmed. Further research would be needed to ascertain all the unknowns regarding fetal microchimerism dynamics in maternal injury and illness.

## Data Availability

The raw data supporting the conclusions of this article will be made available by the authors, without undue reservation.
