# Supplementary material for: Presence of fetal microchimerisms in the heart and effect on cardiac repair
Source: Front Cell Dev Biol. 2024 Aug 5;12:1390533. doi: 10.3389/fcell.2024.1390533 (PMC11350564; doi:10.3389/fcell.2024.1390533)
Supplement: Supplementary file 1 [file DataSheet1.DOCX]

Supplementary Material

# Supplementary Methods

For flow cytometry assays, blood samples (250µL) were collected from C57BL6/J mice under anesthesia (N=1) and stored in MiniCollect tubes with EDTA to prevent coagulation. 20µL of blood were stained with 4′,6-diamidino-2-phenylindole (DAPI) (D9542, Sigma Aldrich) and examined through confocal microscopy to check for fluorescence. An erythrocyte lysis protocol was carried out using Red Blood Cell Lysis solution (RBCL, cat#130-094-183, Miltenyi Biotec). Briefly, 100µL of blood were mixed with 900µL of RBCL and incubated for 10 min at room temperature (RT). Samples were centrifuged at 300xg for 5 min, and the supernatant was removed, obtaining an erythrocyte-free pellet. Samples were analyzed using a MACSQuant Analyzer 16 flow cytometer (Miltenyi Biotec) and images were processed with the Kaluza software.

For the confocal microscopy study, mice were subjected to a transcardial perfusion with Phosphate-Buffered Saline (PBS 1x) to flush out the blood, followed by 4% Paraformaldehyde (PFA) in PBS 1x, and organs were extracted and post-fixed in PFA 4% overnight (o.n.). Organs were embedded in 2% agarose gel blocks and serial sections (80µm) were cut using a vibratome.

Sections were labelled with DAPI as a fluorescent marker for cell nuclei (1:2000, D9542, Sigma Aldrich) in PBS 1x containing 0.1% Triton X-100 for 30 minutes at room temperature (RT). Samples were washed, mounted onto microscope slides alongside fluorescence mounting media (Dako Agilent), and cover slipped.

Samples were analyzed with a Leica TCS-SPE confocal fluorescence microscope. Images of the samples were acquired and processed with the Fiji image processing software.

# Supplementary Figures


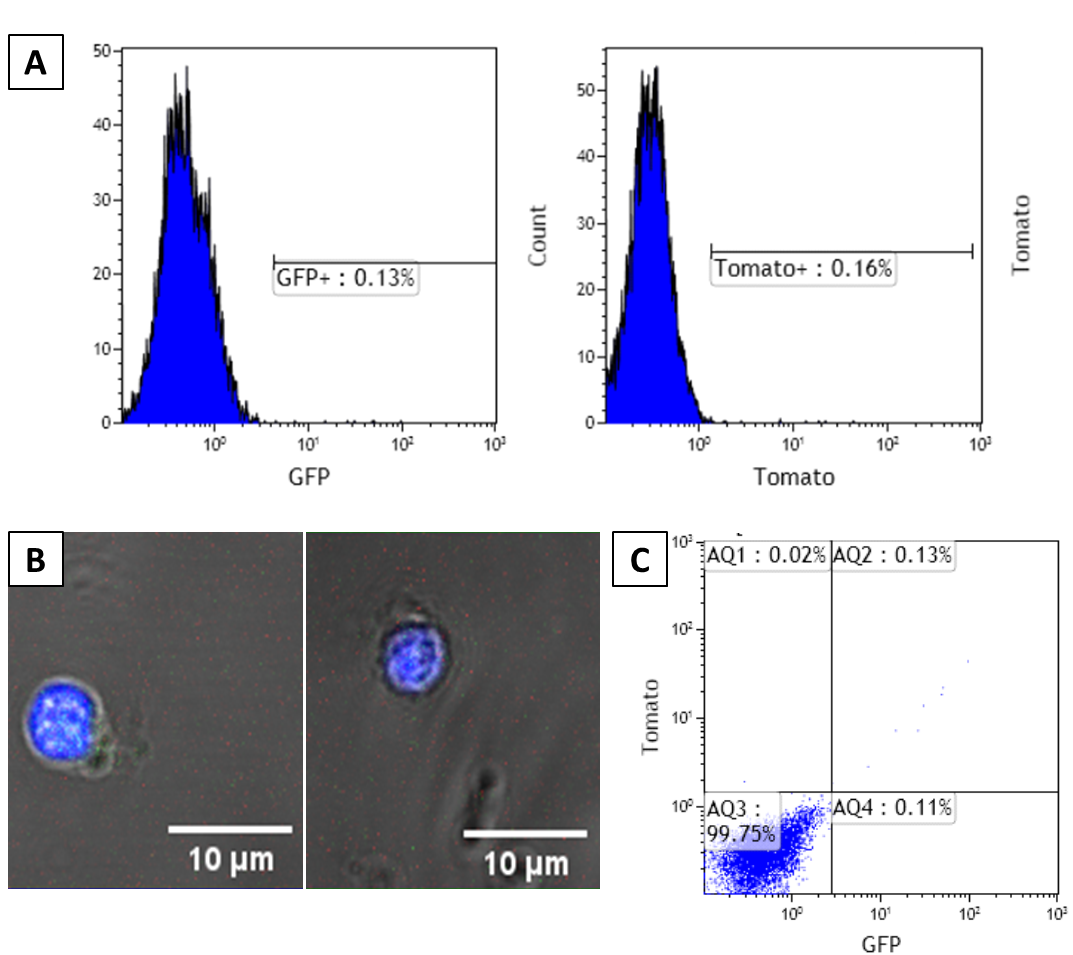


Supplementary Figure 1: Combined flow cytometry and confocal microscopy analysis results for the C57BL6/J WT blood sample. A) Number of fluorescence-positive cells for EGFP and tdTomato. B) Merged fluorescence + brightfield confocal microscopy images of WT phenotype cells. Blue: DAPI. 40x magnification confocal microscopy. C) Fluorescence positivity plot, with 99.75% of all cells testing negative for either fluorophore.


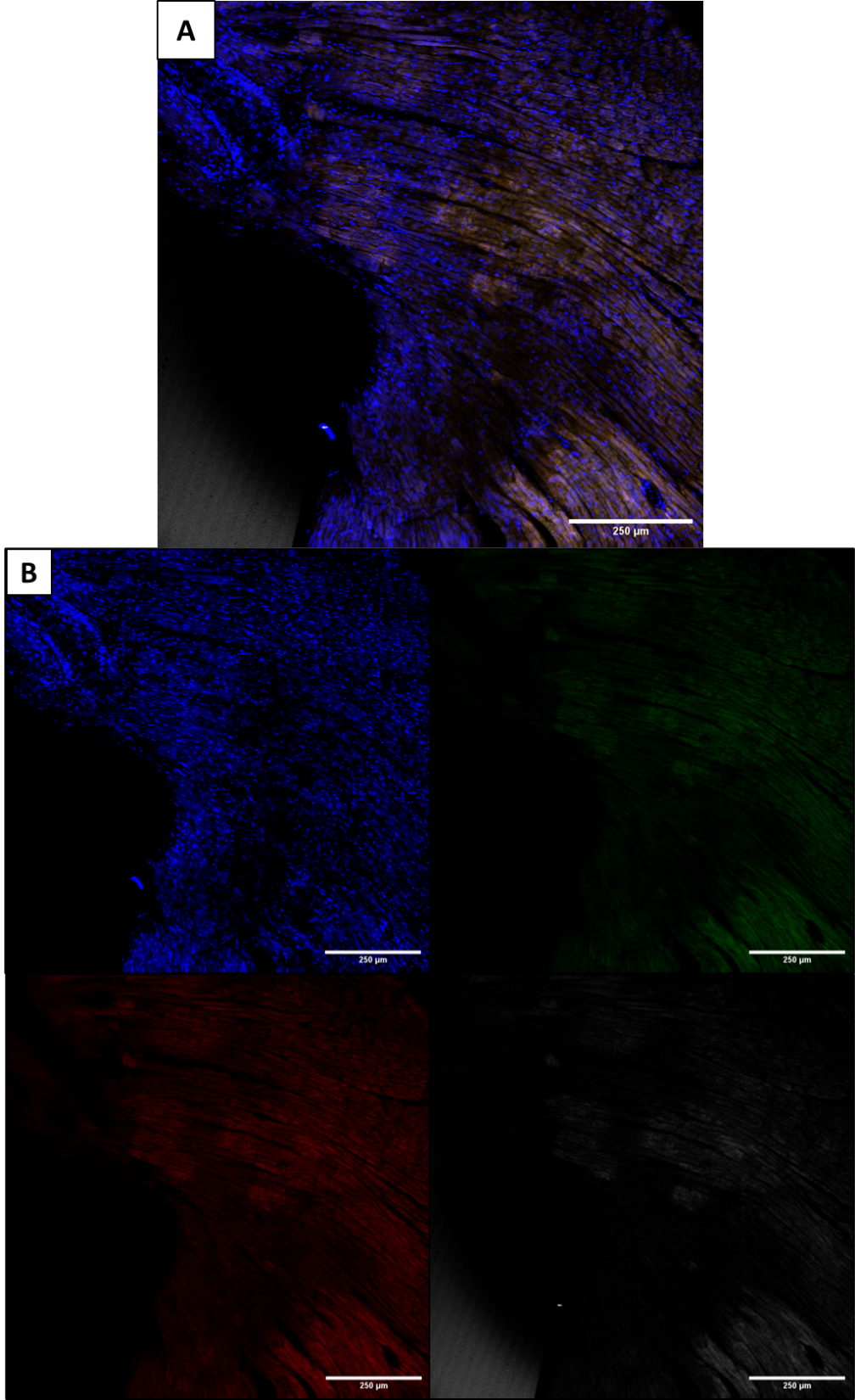


Supplementary Figure 2: Representative confocal microscopy images of female C57BL6/J mice heart samples labelled with DAPI, showcasing no specific fluorescence signal and only autofluorescence in the red, green and far-red channels. Blue: DAPI, Green: EFGP, Red: RFP/tdTomato, Gray: Far Red. A) Multi-channel image of tissue visualization, B) channel breakdown of the image, showcasing specific signal for DAPI and autofluorescence only in the other channels. 10x magnification confocal microscopy.


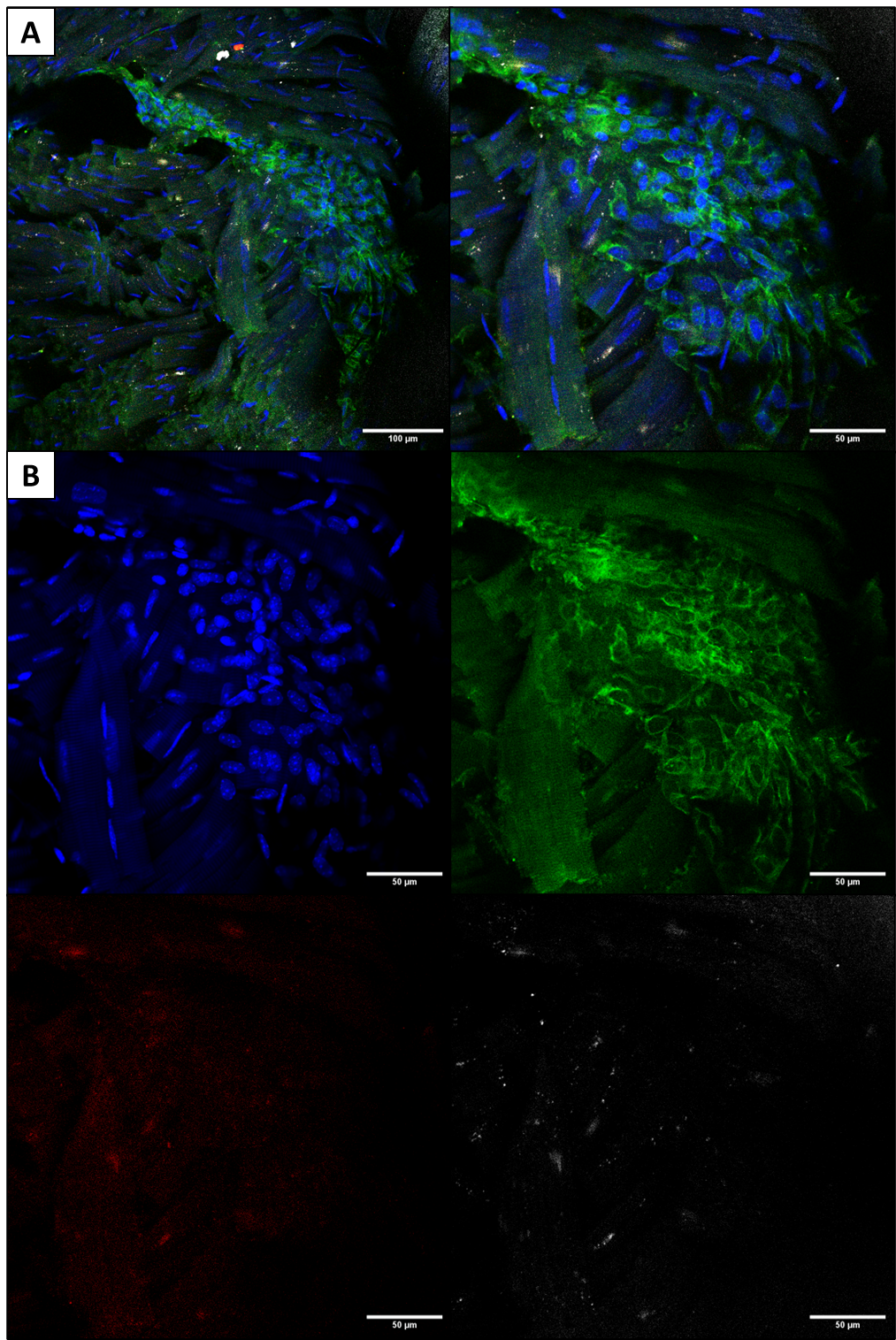


Supplementary Figure 3: Representative confocal microscopy images of postpartum female C57BL6/J WT x ROSA26R-EGFP-Sox2 mice heart samples, showcasing a cluster of EGFP+ cells in the myocardial ventricular wall identified as fetomaternal microchimerisms due to endogenous fluorescence protein expression. Blue: DAPI, Green: GFP/EGFP, Red: tdTomato/RFP, Gray: Far Red. A) Cluster overview, 10x and 20x magnification. B) Channel breakdown of the image, with low/no autofluorescence present in the Red and Far-Red channels, and specific signal in the Green (EGFP) channel.
